# Behaviour and Microstructural Characteristics of Lime-GGBS-Treated Kaolin Clay Contaminated with Gypsum

**DOI:** 10.3390/ma16020874

**Published:** 2023-01-16

**Authors:** Jeremiah J. Jeremiah, Samuel J. Abbey, Colin A. Booth, Anil Kashyap

**Affiliations:** 1 School of Engineering, College of Arts, Technology and Environment, University of the West of England, Bristol BS16 1QY, UK; 2 Centre for Architecture and Built Environment Research (CABER), College of Arts, Technology and Environment, University of the West of England, Bristol BS16 1QY, UK; 3 Office of the Director General, NICMAR University, Pune 411045, India

**Keywords:** gypseous soils, sulphate-induced expansion, lime treatment, GGBS, stabilisation, ettringite, swell, by-products materials, CBR, road-base, sub-base

## Abstract

In this experimental study, the physico-mechanical and microstructural properties of sulphate-bearing clays have been investigated. Sulphate bearing soils constituted by mixing kaolin and gypsum at 0%, 15%, 25%, and 35% gypsum contents were treated with 12% ordinary Portland cement (OPC) and 4%Lime (L) and 8% ground granulated blast furnace slag (GGBS) and subjected to compaction, swell, unconfined compressive strength (UCS), California bearing ratio (CBR), and scanning electron microscopy (SEM) and energy dispersive spectrometry (EDX) analyses. The results of the study showed that the use of L-GGBS improved the soaked CBRs of the treated samples by over 43% when compared to OPC-treated samples after 7-days curing. A reduction in water absorption by 82% was also observed with L-GGBS treatment after 28-days curing. The UCS results also showed better performance with L-GGBS treatment exceeding 856% at 28 days. The effect of increased cementitious product with increasing gypsum content was negated by simultaneous and rapid growth of ettringite minerals which reduced the strength and increased swelling of OPC treated samples up to 18.92%, exceeding allowable limits of 2.5% as specified in Highway Agency Advice Note HA 74/07. The L-GGBS treated gypseous soil samples meet the strength requirement for stabilised sub-base (CS) and stabilised road-bases (CB1 and CB2) as described in TRL ORN31. Hence, the use of L-GGBS combination was found to be effective in ameliorating sulphate-induced expansion and therefore encouraged in the stabilisation of subgrade and road-base materials with high sulphate contents.

## 1. Introduction

There is an ever-increasing need for proper characterisation of the engineering behaviour of stabilised soils underlying road pavements and other civil engineering structures. The safety and durability of facilities sited on engineered earth materials are a direct function of correct modeling of such materials’ behaviour. The application of cement-bound earth materials as road pavement layers have been widely undertaken, following its first successful use in the USA in 1935 [1]. Engineers have continued to explore the use of several natural and man-made binders for the stabilisation of weak soils [2,3,4,5,6,7,8,9,10,11,12,13]. However, ground improvement of sulphate-rich soils using cement and lime have been undertaken in the past with resultant expansion and dessication cracks following precipitation of expansive minerals such as thaumasite and ettringite crystals synonymous with aluminate-sulphate reactions in the presence of calcium oxide [14,15,16,17]. The adverse effects of sulphate content on cement and lime-stabilised materials have reflected various attributes of the treated soils such as lower strength, increased permeability, and volume instability [18]. Additionally, the effects of external sulphate attack from contaminated ground water ingress into cement and lime stabilised soils may exacerbate these concerns with increasing sulphate content [19].

**Figure 1:**
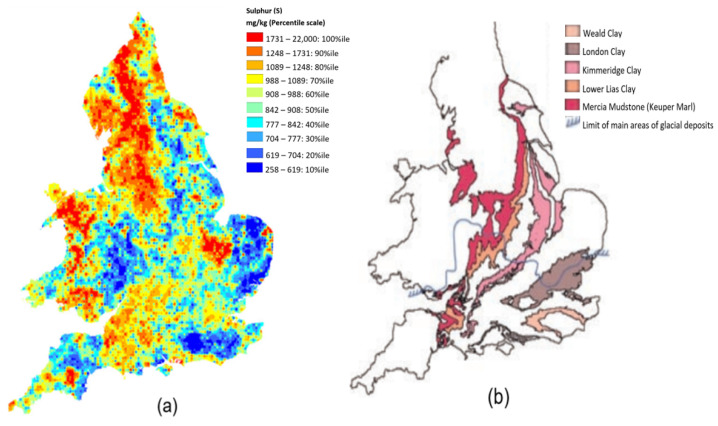
(**a**) National Soil Inventory Topsoil Sulphur Concentrations [20]. (**b**) Major Sulphate/Sulphide-bearing Strata in England and Wales [19].

Sulphates are common in soils containing salts due to the oxidation of sulphides. These soils are common in the UK following weathering of ancient strata of clay, shale, and mudstone deposits from the carboniferous time-periods [20]. Figure 1a shows a distribution of sulphur content in mg/kg, while Figure 1b shows the major sulphate and sulphide-bearing strata across the UK. The Figures reinforce the need for adequate field investigation to accommodate sufficient sulphide/sulphate content appraisal prior to the commencement of cement or lime stabilisation. This guidance is already enshrined in the Highway Agency Advice Note HA 74/07 for cement and/or lime treatment of fill and capping materials [21].

The hydration process leading to the formation of strength-giving compounds, such as calcium silicate hydrates (CSH) and calcium aluminate hydrate (CAH), simultaneously produces expansive minerals like ettringite [Ca_6_Al_2_(SO_4_)_3_(OH)_12_⋅26H_2_O] in the presence of sulphate (SO_4_^2-^), as expressed below [22].

6Ca^2+^ + 2Al(OH)_2_^−^ + 4(OH)^−^ + 3(SO_4_)^2−^ + 26H_2_O → Ca_6_Al_2_(SO_4_)^3^(OH)_12_⋅26H_2_O (1)

These expansive minerals are susceptible to large volume changes up to 2.5 times the soil volume with sufficient moisture [23,24,25] and are most detrimental to light-weight structures [26]. Earlier, the complexities of the contributing factors to swelling and expansion of cement and lime treated sulphate-bearing soils have necessitated several investigations, including assessing the mechanical and chemical properties of these soils under various conditions, namely sulphate contents, curing periods, curing temperatures, and compaction efforts to mention a few. A study by Kampala and others [18] examined the effects of sulphate content on strength and compaction characteristics of cement-treated sulphate soils using clay-sodium sulphate mixtures. The study showed that sulphate content had minor effects on the density-moisture behaviour of compacted cement-treated sulphate-bearing soils but led to strength depreciation even for samples treated with higher cement contents and longer curing durations compared to non-sulphate samples due to the calcium-aluminate-sulphate products [18]. The effects of sulphate-rich hydration products were found to reduce the compressive strength of OPC-treated artificially constituted kaolin-gypsum system by 47% [27] and can be partly linked to increased moisture absorption of the expansive minerals with increasing sulphate content [28]. It can be concluded that the presence of ettringite needles within the micropores of cement and lime treated soil fabrics negate the cementitious effects of the strength yielding minerals [14,16]. Similarly, a large volume change of up to 7% was observed by Caselles and others [29] in a lime-treated sulphate bearing soil containing 1 wt.% of sulphate while 6.1% expansion was reported in a stabilised kaolinitic soil with 2 wt.% sulphate content [30]. Such large volume changes are detrimental in pavement subgrade and base layers with associated economic penalties.

This deleterious impact has informed the evaluation of alternative binder combinations in combating sulphate-induced expansion in sulphate-rich soils where the total potential sulphate (TPS) exceeds allowable values [31,32]. According to industry guidelines such as [21], these limiting values need to be evaluated on a site-specific basis to determine the peculiar behaviour of treated soils in order to ascertain the suitability of such soils for lime or cement stabilisation, and if alternative binders would be appropriate to mitigate adverse effects. To this end, several researchers have explored various binders. For instance, Adeleke and others [33] utilised MgO and GGBS in stabilising a high sulphate-bearing soil and reported that magnesium-based cementitious products enhanced the physical and mechanical properties of the treated soils by reducing swell and improving the UCS over OPC-treated counterparts. Bazyar [34] investigated the suitability of lime and rice husk ash (RHA) in improving the strength and reducing the swell potential of a natural gypseous clay and reported that the addition of RHA significantly ameliorated sulphate-induced heaving with optimum performance at 6%–8% and 8%–10% of lime and RHA respectively. Pulverised fuel ash (PFA) has also been utilised in surpressing ettringite-induced swelling of lime-treated sulphate-bearing clays by the formation of new hydration products which tend to inhibit sulphate expansion and also reduced the plasticity index of the treated soils [22]. An experimental investigation by Puppala and others [35] considered PFA, GGBS, lime, polypropylene fibre, and sulphate-resistant cement in stabilising sulphate-bearing subgrades. Puppala and others [35] showed that there were significant improvements in the mechanical properties of GGBS and PFA-treated sulphate samples. However, the combination of lime and polypropylene fibre was reported to have performed better in suppressing volume instability and improving the UCS of the treated soils. Other studies have utilised alternative materials such magnesium oxide [36].

Additionally, the inclusion of GGBS with other binders such as lime has been shown by several laboratory and field trials to ameliorate sulphate induced heave by immobilizing dissolved sulphates in the alkalinized pore fluids of treated soils [29,37,38]. A study by the Texas department of transportation showed that lime-GGBS binder combination was able to suppress swell while simultaneously increasing strength of treated sulphate soils [39]. Furthermore, like other industrial and agricultural residues, the use of GGBS lessens the already heightened concerns of increasing landfill wastes synonymous with the production of the more common traditional calcium-based stabilisers (CBS). In addition, the use of GGBS is known to be advantageous as an eco-friendlier stabiliser with lower carbon-footprint than CBS [40,41,42,43].

While the combination of lime and GGBS has been utilised for stabilisation of gypseous soils [16,38,44,45,46,47], there is still insufficient information on vital parameters such as soaked and unsoaked CBR, stiffness modulus, and microstructural characteristics of LGGBS treated highly gypseous soils. This study expands this knowledgebase by evaluating and comparing the performance of both OPC and L-GGBS in treating several gypseous clays with a focus on the major parameters relevant to road pavement design and construction such as UCS, soaked and unsoaked CBR, stiffness modulus, swell, water absorption, and microstructural characteristics of OPC and L-GGBS stabilised gypseous soils. This study evaluates the performance the stabilised material in terms of the limiting requirements for stabilised roadbase and subbase for road pavement construction.

## 2. Materials and Methods

### 2.1. Materials

The materials used in this experimental work include kaolin(K), gypsum (GYP), lime(L), and ground granulated blast furnace slag (GGBS). Gypsum (CaSO_4_⋅2H_2_O), a product of the reaction of calcium carbonates and sulphuric acid (H_2_SO_4_), can be used to constitute sulphate-bearing soils in the laboratory [29,33,48]. The OPC used in this study is CEM1. The gypsum, lime, and GGBS used in this study have been used in a previous study [48], with the lime in conformity with [49]. The chemical compositions of the materials are given in Table 1 below.

### 2.2. Experimental Design and Sample Preparation

The gypseous soils were constituted by thoroughly mixing dry kaolin with various gypsum contents by the mass of the dry soils until a homogeneous distribution was obtained. To study a wide range of gypseous soil behaviour, a total of 4 gypseous soils were constituted with gypsum contents of 0%, 15%, 25%, and 35 wt.% of dry soil, translating to 0, 7, 11.8, and 16.5 wt.% of sulphate, respectively, and are classified by Barazanji [50] as slightly to highly gypseous soils, as shown in Table 2.

The compaction characteristics (MDD and OMC) of the artificially synthesised gypseous soils were determined using the Standard Proctor compaction method according to [51]. The consistency limits of the gypseous soils were also determined in line with [52] and presented in Table 3. It was observed during the syntheses of the gypseous soils, that the addition of gypsum to kaolin reduced the plasticity index by reducing the liquid limit of the gypseous soil with increasing gypsum content, as shown in Table 3.

**Figure 2:**
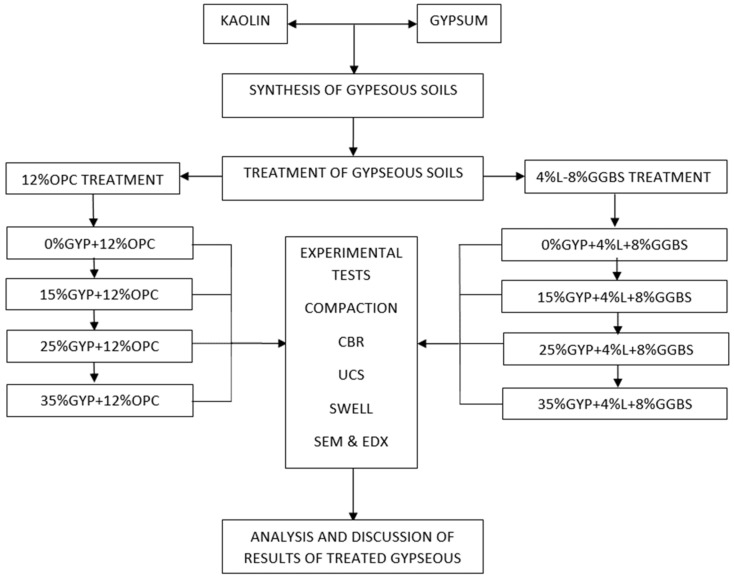
Experimental flowchart.

The OPC treated samples were prepared by mixing dry kaolin with gypsum and allowed for 24 h before applying 12%OPC treatment and compacting, as stated earlier. For the L-GGBS treated soils, samples were prepared by mixing pre-constituted gypseous soils first with 4%lime and left for 24 h to allow for mellowing, after which 8%GGBS by weight of dry gypseous soil was added and mixed thoroughly to ensure homogeneity. The addition of lime before GGBS has been suggested by Higgins [53] to be suitable for the treatment of cohesive soils. The treated gypseous soils were then mixed with distilled water and compacted on the wet-side of optimum at 110% of the OMC of the untreated gypseous soils by gradual addition of the pre-determined mass of water while continuously mixing the soil to ensure uniform wetness for each batch. Three samples were prepared for each mix ratio and curing period for both 7 and 28 days curing for UCS, 2 samples were prepared for each mix ratio for Soaked and Unsoaked CBR. Samples were also prepared for swell, SEM, and EDX tests following the experimental flow chart shown in Figure 2.

**Figure 3:**
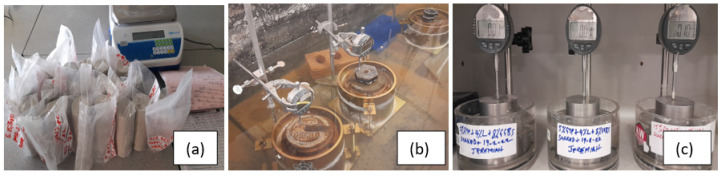
Treated gypseous soil specimens. (**a**) UCS samples. (**b**) Soaking of CBR samples. (**c**) Swell testing.

Furthermore, to evaluate the effect of 12%OPC and 4%L-8%GGBS treatment on the compaction characteristics of the treated gypseous soils, the seven days cured OPC and L-GGBS-treated gypseous soils were air-dried at room temperature of 22 °C for 14 days and then pulverised, remixed, and tested for compaction as before. The details of the mix compositions used in sample preparation is given in Table 4. For UCS tests, a total of 72 cylindrical test samples of 50 mm diameter and 100 mm depth were prepared in line with [54]. The UCS samples were sealed in polyethylene bags to prevent moisture loss during curing, as shown in Figure 3, and cured at room temperature of 22 °C for the specified periods before testing in a load frame.

For the CBR samples, a total of 96 cylindrical samples of 152 mm diameter and 127 mm height were prepared for both soaked and unsoaked CBR test. The CBR samples were also cured while preventing loss of moisture according to [55] before soaking in water at 20 ± 2 °C for 96 h prior to testing, as shown in Figure 3b. The unsoaked CBR samples were tested at the end of the curing period without soaking.

The vertical swell of the CBR samples during soaking was measured with dial gauges and recorded. The weight of the treated soils was also measured before and after the soaking period and used to determine the water absorption of the samples.

### 2.3. Experimental Testing

#### 2.3.1. Unconfined Compressive Strength Test

**Figure 4:**
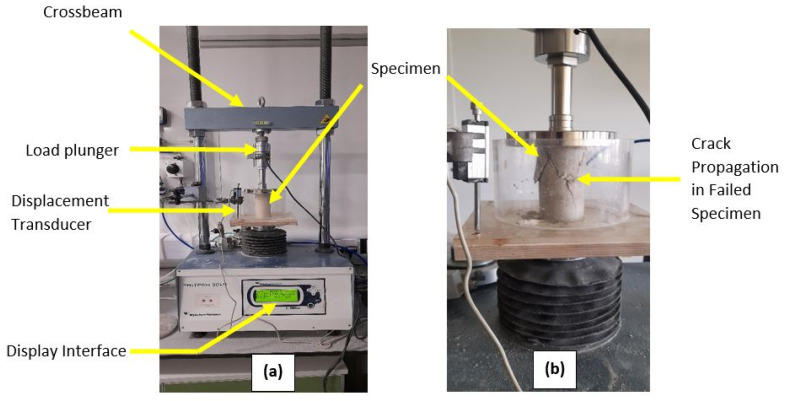
Gypseous soil sample in UCS testing. (**a**) UCS Test Set-up (**b**) Failed Sample in UCS Test.

UCS was conducted on cured cylindrical samples using in a VJ Tech 50 kN triaxial load frame in accordance with [56]. Three samples were tested for each mix ratio in line with [54] and the mean UCS was determined for analysis. The UCS was obtained as the ratio of the applied load to the cross-sectional area of the cylindrical samples. The samples were loaded at a strain rate of 1 mm/min while automatically logging the applied force and sample deformation to a dedicated computer system. The samples were then loaded to failure as shown in Figure 4 or until a 15% strain was attained at which point unshared samples were deemed to have failed.

#### 2.3.2. California Bearing Ratio Test

The CBR penetration test was conducted on 150 mm diameter × 127 mm depth samples in accordance with [55] using the base side of the samples. All samples were penetrated using a VJ Tech 50 kN load frame until the measured force began to decline indicating failure or a maximum of 10 mm penetration was attained. Chunks of soil from the middle of failed samples were collected for SEM and EDX analyses.

#### 2.3.3. Swell Test

The effects of the Treatments were measure by conducting swell test on treated gypseous soils. Samples were soaked in water under a surcharge of mass 930 kg while measuring the vertical deformation. The swelling of the samples was recorded for 56 days and expressed as a percentage of the original height of the samples.

#### 2.3.4. Scanning Electron Microscopy and Energy Dispersive Spectrometry

To investigate the microstructural characteristics and mineralogical composition of the soil samples, SEM and EDX studies were carried out on chunks of stabilised samples. The soil samples for SEM and EDX analyses were taken from the middle sections of large CBR samples in order to obtain better representative samples for microstructural study following treatment. The samples were gold-coated in an Emscope SC 500 unit within 1 min using a current of 7.5 mA at a vacuum pressure reading of 0.1 Torr during coating. Samples were glued with a Leit-C conductive carbon cement and allowed for 48 h to dry prior to analysis. The samples were put in a FEI QUANTA FEG 650 SEM Unit at a chamber pressure of about 9.5 × 10^−7^ Torr and an accelerating voltage of 5–20 kV [57]. The micrographs were taken at varying levels of the stage at 10–20 mm depending on the size of the sample. EDX was utilised in evaluating the oxide composition and concentration.

## 3. Results and Discussion

### 3.1. Compaction Characteristics

**Figure 5:**
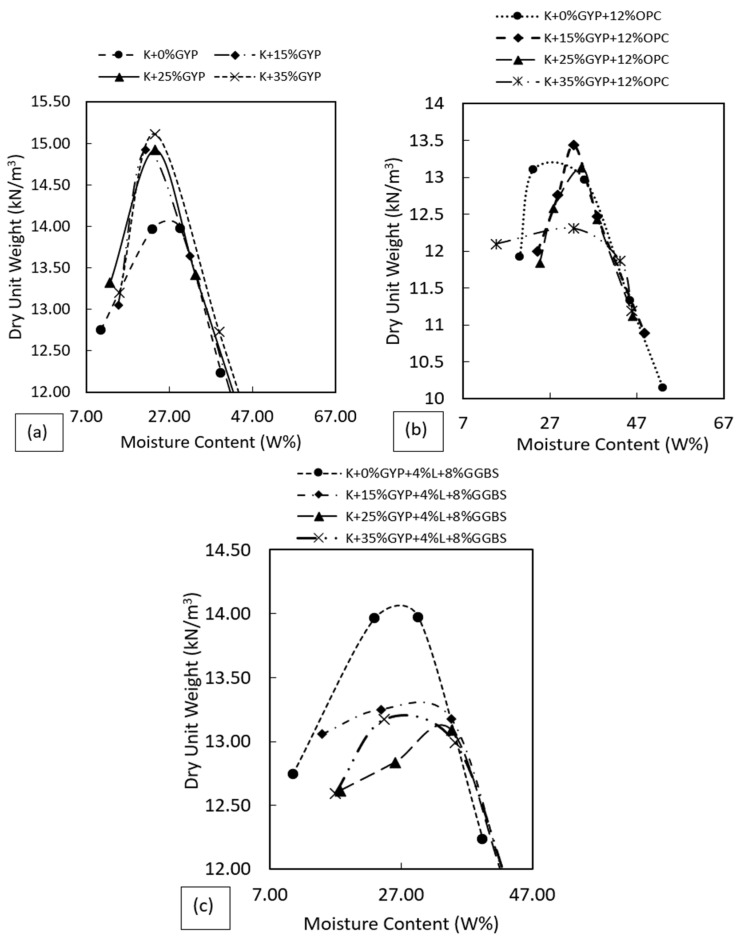
Compaction curves of the treated and untreated gypseous soils. (**a**) Untreated gypseous soils. (**b**) 12%OPC treated. (**c**) 4%L and 8%GGBS treated.

The results of the standard Proctor compaction test on the treated and untreated artificially synthesised gypseous soils are presented in Figure 5. The results show that the addition of gypsum increased the MDD of the untreated gypseous soils with reducing OMC. This may be linked to the reduced pore spaces following interstitial gypsum particles within the micropores of the kaolin-gypsum mixture, which increased the mass per unit volume of the gypseous soils. Gypsum addition reduced the affinity of the kaolin-gypsum mixture for water which is seen in the reduced plasticity index following reduced liquid limits. The reduction in OMC allowed for a more compact system to be obtained at lower moisture levels leading to a denser material with increasing gypsum content, as shown in Figure 5a.

The effect of 12%OPC and 4%Lime-8%GGBS on the compaction characteristic of the gypseous soils is shown in Figure 5b,c. Overall, the addition of 12%OPC and 4%L-8%GGBS resulted in a reduction in the MDDs of the treated gypseous soils. The reduction could be linked to flocculation and agglomeration of clay particles which increased the voids. Large-sized agglomeration impeded the effect of compaction at the same applied compaction effort which reduced the dry densities. This reduction in dry density of some treated fine grain soil is also reported in previous studies utilising both cement and lime treatments [17,58,59,60]. A reduction in dry density with increasing gypsum content was observed for the gypseous soils with higher gypsum content for both OPC and L-GGBS treated samples. Higher gypsum contents in addition to the treatment additives resulted in excessive binder content, which increased the amount of unreacted cement and gypsum particles leading to a reduction in the dry density of the treated gypseous soil, as shown in Figure 5b,c. However, the dry density of the L-GGBS-treated gypseous soils was observed to be higher than that of the OPC-treated soils. The higher densities show better performance of the L-GGBS combination in enhancing the dry density and overall strength of the L-GGBS treated soils over OPC treated soils, which is desirable in road pavement applications as the shear strength is greatly influenced by the MDD [61].

### 3.2. Swell

**Figure 6:**
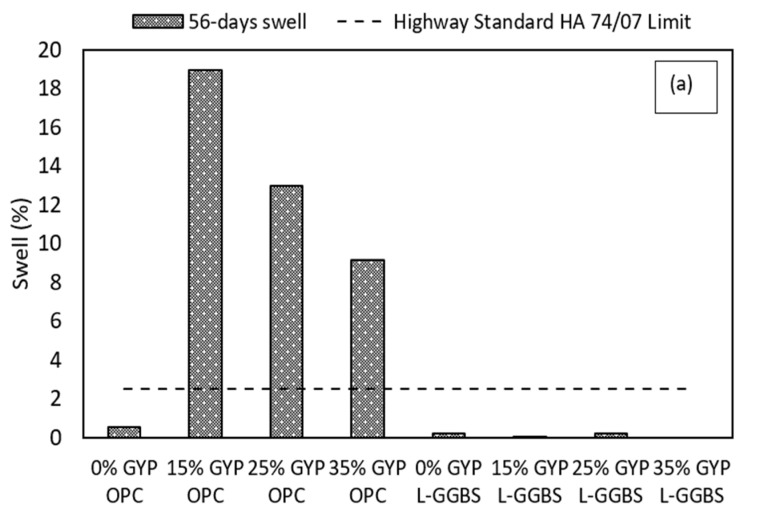

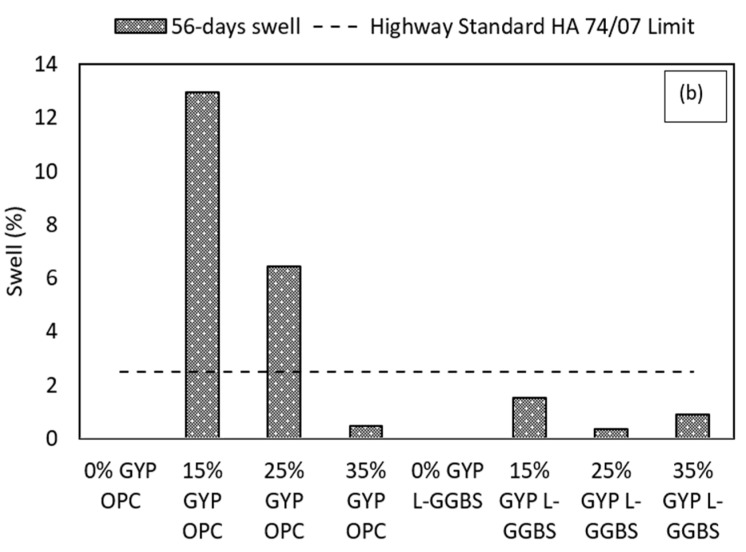
Vertical swell of OPC and L-GGBS-treated gypseous soils. (**a**) 7-days cured. (**b**) 28-days cured.

Swelling of OPC treated sulphate bearing soils is an unwanted behaviour, especially underneath light-weight structures such as road pavements. OPC treatment of sulphate-bearing soils has been shown to result in significant swelling and expansion when subjected to water ingress [1,62,63]. This behaviour leads to the development of early tension cracks in heaved sections of flexible pavements. To evaluate the swell response of the OPC and L-GGBS-treated gypseous soils, vertical swell tests were conducted on stabilised samples. Figure 6 shows the swell results of the treated soils after 56 days soaking in comparison to significant swell benchmarks [64] and the Highway Standards as specified in [21] and shown in Table 5. From the results, the swell of all gypseous soil samples treated with 12%OPC increased rapidly within the first 10 days and eventually exceeded the limit of 2.5% specified in [21].

The 15% OPC-treated gypseous soil showed the greatest swelling of 18.92% and was greater than that of the 0% gypseous soil and maybe is attributed to combined gypsum crystal expansion and ettringite-induced heave [65]. However, OPC-treated samples with higher gypsum contents showed a relatively slower expansion rate due to increased CaO available for precipitation of more binder gels (CSH gels) with increased bonding from the reaction of both gypsum and cement particles as expected [27]. A maximum swell of 12.96% and 9.16% was observed for both 25% and 35% gypseous soils stabilised with OPC at the end of the 56 days soaking period. It can be noticed from the SEM results of the microstructural features in Section 3.6 that a more compact and dense soil morphology is achieved with higher gypsum content with reduced pore sizes. Furthermore, higher gypsum contents, especially for the 25% and 35% gypsum contents, may have resulted in a higher percentage of unreacted gypsum and OPC particles within the pores of the treated gypseous soil systems, impeding expansion and further forming ettringite crystals by slowing down the hydration reaction of Ca^2+^-Al^3+^-SO_4_^2−^ crystals leading to a slower swell rate and percentage [66,67].

However, the L-GGBS treated gypseous soils showed much slower expansion rate and overall reduction in total volume change at the end of the 56 days soaking. A maximum swell of 0.2% and 0.04% respectively was observed in 0% and 15% gypseous soils treated with L-GGBS. The L-GGBS-treated sample containing 25% gypsum showed only 0.2% swell while that of 35% gypsum content had 0% swell at the end of 56 days soaking period. The combination of Lime and GGBS treatment enhanced the performance of treated soil under soaked condition. This indicates that, although the formation of the strength-yielding compounds was accompanied by the development of ettringite minerals, the associated expansive tendency of the crystals in the presence of water was significantly reduced when compared to that of the OPC-treated samples. Similar results from L-GGBS treated sulphate soils have been reported in both laboratory studies and field trials [68,69]. The 28 days swell behaviour of the treated samples was very different for both OPC and L-GGBS treated samples when compared to the seven days cured samples. The 28 days cured samples all showed reduced swell rate and overall reduction in swell, as shown in Figure 6b. The reduction in swell potential of the 28-ays cured samples could be related to a much stronger bond between soil particles following a longer curing period [30]. However, the OPC treated samples showed higher swell than the L-GGBS treated samples suggesting that L-GGBS treatment might be a better option in ameliorating sulphate-induced heave in subgrade materials.

### 3.3. Water Absorption Characteristics

Water absorption characteristic is useful in evaluating the performance of treated gypseous soils in the event of harsh environmental conditions such as flooding which could lead to rapid break-down of the soil-binder fabric and eventual strength depreciation. The effect of the binders in reducing the pore spaces and cutting down on the amount of water absorbed was studied following 96h soaking of treated and untreated CBR samples. The water absorption characteristics of the gypseous soils show that both OPC and L-GGBS are effective in reducing the water absorption propensity of the stabilised soils when compared to the untreated gypseous soils as expected [67].

**Figure 7:**
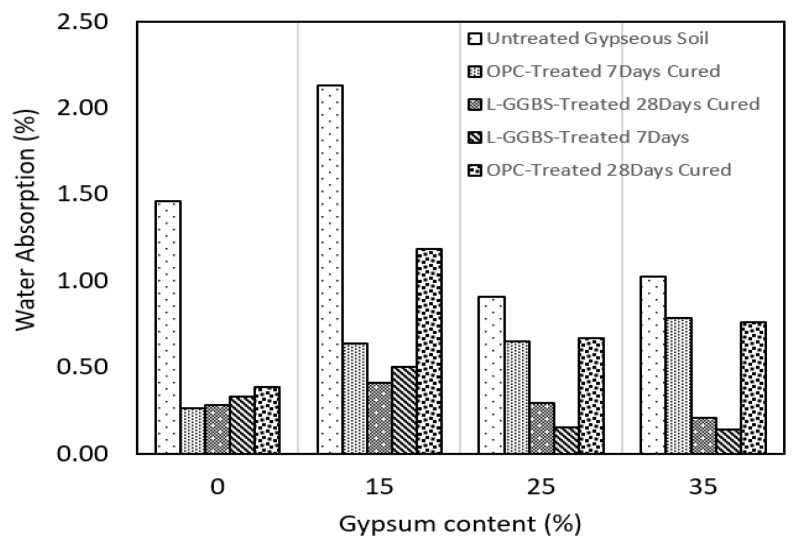
Water absorption of treated and untreated gypseous soils.

For the untreated gypseous soils, the highest water absorption was observed in the 15% gypseous sample and could be linked to a rapid dissolution of gypsum particles within soil pores during soaking. The lower gypsum content of the 15% untreated gypseous soil resulted in poor cementitious effects which was worsened by soaking, leading to increased water absorption, as shown in Figure 7. This behaviour is in alignment with the swell curves presented in Section 3.2.

The water absorption of the gypseous soils with higher gypsum content was observed to be lower for both Untreated, OPC, and LG treatment. This is a direct effect of increased cementation with the known effect of sealing pore spaces with cementitious gel, increasing density, and reducing soil permeability [70]. The reduced water absorption of the untreated gypseous samples indicates that the presence of gypsum might have induced slight resistance against water ingress. This behaviour of the untreated gypseous has been highlighted in earlier research reporting on the effectiveness of gypsum as a stand-alone binder and in combination with lime in improving the strength and reducing the water absorption of some treated soils [71]. However, further reduction in the water absorption of the gypseous soils was observed following OPC and L-GGBS treatment, as shown in Figure 7.

The water absorption of seven days-cured OPC-treated samples, as shown in Figure 7, was lower than that of the untreated gypseous samples, depicting improved bonding and cementation. In particular, the 15% untreated gypseous soil with water absorption of 2.2% reduced to about 0.60% following cement treatment. This value reduced further to 0.40% for the L-GGBS-treated counterpart. The OPC treated gypseous soils with higher gypsum content showed lower absorption of water indicating a denser soil matrix compared to the untreated gypseous soils. L-GGBS treatment showed the most effect in terms of resistance to water absorption. A reduction in water absorption of 82% was observed for the 15% gypseous soils treated with L-GGBS and cured for 28 days. Additionally, the water absorption of the L-GGBS treated gypseous soils with 25% and 35% gypsum content was reduced by 69% and 80% respectively. This reduction in water absorption is a significant improvement to both the untreated and OPC-treated samples and has been reported in other studies involving lime and gypsum treatment [70]. The swell result shows that L-GGBS treatment might be better in reducing pore spaces and overall water absorption of the gypseous soils.

### 3.4. Unconfined Compressive Strength and Stiffness Modulus

**Figure 8:**
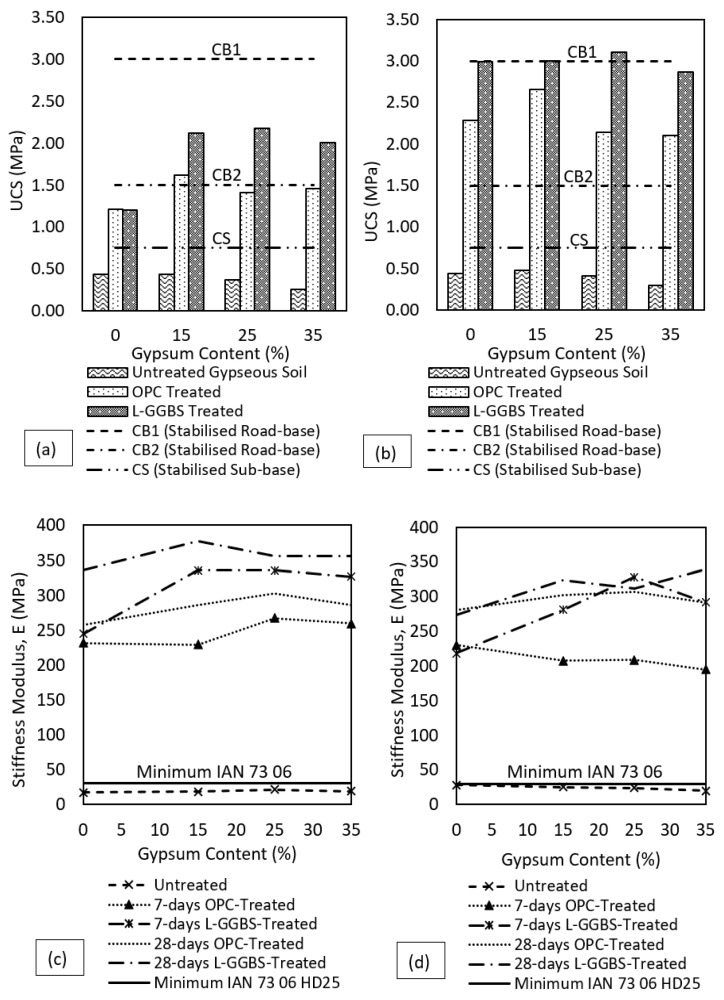
UCS of OPC and LG treated gypseous soils. (**a**) 7-days cured. (**b**) 28-days cured. (**c**) Soaked stiffness modulus. (**d**) Unsoaked stiffness modulus.

The results of UCS tests on both treated and untreated gypseous soils show that the strength of the gypseous soils were significantly improved following OPC and L-GGBS treatment, as shown in Figure 8. Treating with OPC as expected resulted in the formation of an enhanced soil-binder system with strengths exceeding that of the untreated gypseous soils. As shown in Figure 8a, the UCS of the untreated gypseous soils were lower in strength when compared to the seven days UCS of the treated gypseous soils. The 7-days UCS of the untreated gypseous soils reduced in strength from 0.44 MPa to 0.26 MPa for 0% to 35% gypsum contents, respectively. Higher gypsum content further reduced the UCS of the untreated soils. The reduction in the UCS of the untreated gypseous soil might be attributed to increased sulphate contents in the absence of additional strength-yielding compounds at OMC, which lowered the UCS of the untreated gypseous soils and has been reported in [27]. Strength gain is mainly due to cementation from calcium-silica-hydrate and calcium-aluminate-hydrate reactions initiated in the soil-binder medium at higher pH, as reported by [48,72,73], which was not the case with the untreated gypseous soils at higher gypsum contents.

Consequently, the OPC-treated gypseous soils exhibited further strength improvement over the untreated gypseous soils based on further precipitation of strength yielding compound namely calcium-silicate hydrates (C-S-H) and calcium-aluminate-hydrates (C-A-H). At seven days curing, the OPC-treated samples had UCS of 1.62 MPa to 1.46 MPa for 15% to 35% gypsum contents respectively. The 7-days UCS of the OPC-treated samples meets strength requirements for stabilised sub-base (CS) applications as stated in [74]. As shown in Figure 8a,b, almost all the gypseous soil samples treated with OPC fall below the CB2 and CB1 strength requirement at seven days curing. However, the gypseous soils treated with L-GGBS showed better strength performance over the OPC-treated counterparts at seven days curing with strength of 2.12 MPa to 2.01 MPa for 15% to 35% gypsum content respectively. All L-GGBS-treated gypseous soil samples exceeded the requirement for CS, and stabilised road-base (CB2) even at seven days curing. For both stabilisers, the strength of the treated gypseous soils declined with increasing gypsum contents. The failure mechanism of the treated soil was characterised by a more brittle failure which as expected, is due to increased shear strength with reduced plastic deformation under loading. The high pH of the alkalised soil-water system from 4%L addition allowed a quick strength gain during the mellowing period through cation exchange and was further enhanced by the addition of 8%GGBS, which improved the UCS of the L-GGBS treated soils above the that of the OPC-treated samples. As expected, the 28-days cured L-GGBS, and OPC-treated samples showed significantly higher strength gain over the untreated soils due to increased cementation and bonding.

The 28-days cured L-GGBS treated samples performed better than the OPC-treated. The treated samples increased in strength as more bonding was achieved with increased precipitation of the strength compounds over time. However, a reduction in UCS was also observed with increasing gypsum content which was like the trend of the 7-days cured samples. Slow strength gain has been highlighted to be synonymous with the use of GGBS and reported in previous studies as shown in [53,75]. However, the UCS of L-GGBS treated samples performed better even at seven days curing as shown in Figure 8. The performance of the 28-days cured samples is shown in Figure 8b. The L-GGBS-treated samples increased in strength by a minimum of 856% when compared with the untreated gypseous samples after 28-days curing. On the other hand, the OPC-treated samples increased by a minimum of 627% when compared with the untreated gypseous samples at 28 days curing. The higher UCS of the L-GGBS treated samples indicates the suitability of the L-GGBS binder combination in dealing with low strengths of highly gypseous soils. The results show that the L-GGBS is a suitable alternative to OPC treatment for pavement construction with the added advantage of a lower carbon footprint compared to OPC treatment [75]. At 28 days curing, while the OPC-treated samples were below CB1 strength requirements, all L-GGBS-treated gypseous soil samples exceeded this strength requirements and proved suitable for CS, CB1, and CB2 applications.

The stiffness modulus of the stabilised soil is presented in Figure 8c,d. The stiffness modulus was estimated based on the empirical relation given by Powell and others [76] and suggested in [77]. The stiffness modulus is a key parameter in the selection of base thicknesses of pavement foundations in the design of flexible pavements. The untreated gypseous soil samples under soaked condition, as expected, showed low stiffness below minimum requirement across all gypsum contents while the treated samples improved significantly for both binders. Under soaked conditions, the stiffness modulus was higher than that of the unsoaked samples. As earlier mentioned, soaking for 96h enhanced the curing process and lead to better bond development which improved the stiffness of the treated soil. As with the UCS result, the stiffness of the L-GGBS treated samples where higher than the OPC-treated samples. The seven days cured L-GGBS treated samples showed higher stiffness than the 28-days cured OPC treated counterparts at higher gypsum contents. This again highlights the advantage of the L-GGBS treatment over the OPC in high sulphate-bearing soils.

### 3.5. California Bearing Ratio

**Figure 9:**
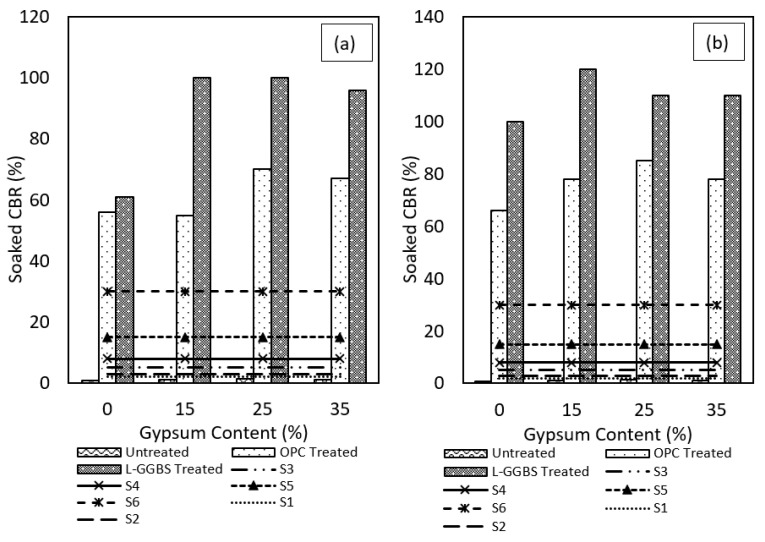
Soaked CBR results for OPC and LGGBS treated gypseous soils. (**a**) 7-days cured. (**b**) 28-days cured.

The performance of treated soils as subgrade and subbase materials in road construction is usually evaluated based on the soaked and unsoaked CBR tests. In the current study, gypseous soil samples stabilised with both 12%OPC, and 4%L and 8%GGBS combination were subjected to 96h soaking and tested for CBR. The untreated gypseous soil was also tested to compare the improvements. Whereas the untreated gypseous soils showed very low penetration resistance following 96h soaking, the stabilised soils showed higher resistance as shown in Figure 9 indicating that both binders increased the CBR of the treated soils under soaked condition. The soaked CBR values of the 7-days cured OPC-treated gypseous soils were less than the L-GGBS treated samples across all gypsum contents. A minimum soaked CBR of 55% was observed at 15%gypsum content for the OPC-treated samples whereas the L-GGBS-treated samples had a minimum soaked CBR of 96% occurring at 35%gypsum content at the end of the soaking period. The results from Figure 9a,b show that for the 7-days cured samples, a maximum soaked CBR value of 70% and 100% for OPC and LGGBS treatment respectively occurring at 25% gypsum content. For the L-GGBS treated samples, for both 7 and 28-days curing durations, a maximum CBR was observed for the 15%gypseous soil. Overall, the 7-days cured L-GGBS-treated samples performed better than the OPC-treated samples. Apparently, the use of L-GGBS improved the 7-days soaked CBRs of the treated samples by 82%, 43%, and 43% for 15%, 25%, and 35% gypsum contents, respectively, when compared to the result of the OPC-treated samples. Additionally, the 28-days cured samples showed higher soaked CBR values for both OPC and L-GGBS than the 7-days cured samples as expected.

The increased CBR of the 28-days cured samples is linked to more strength gain because of increased precipitation of the strength-giving compounds over a longer hydration period. Like the 7-days cured samples, the results of the 28-days cured samples showed better performance with L-GGBS treatment than OPC-treatment. Samples with higher gypsum contents showed a slight reduction in soaked CBR and may be linked to excessive unreacted gypsum and OPC or L-GGBS particles within the matrix of the treated soils. Upon soaking these particles will dissolve in the pore fluid and contribute to lower CBRs. This behaviour indicates that an optimum gypsum-binder mix ratio for maximum penetration resistance under soaked conditions may exist and requires further investigation. Overall, the gypseous soil samples stabilised with 4%L and 8%GGBS showed better performance by offering the most resistance against penetration. This corroborates earlier conclusions from previous studies that the L-GGBS combination is effective in maintaining the strength of the inter-particle bonds of the treated soils under soaking conditions [78,79,80].

**Figure 10:**
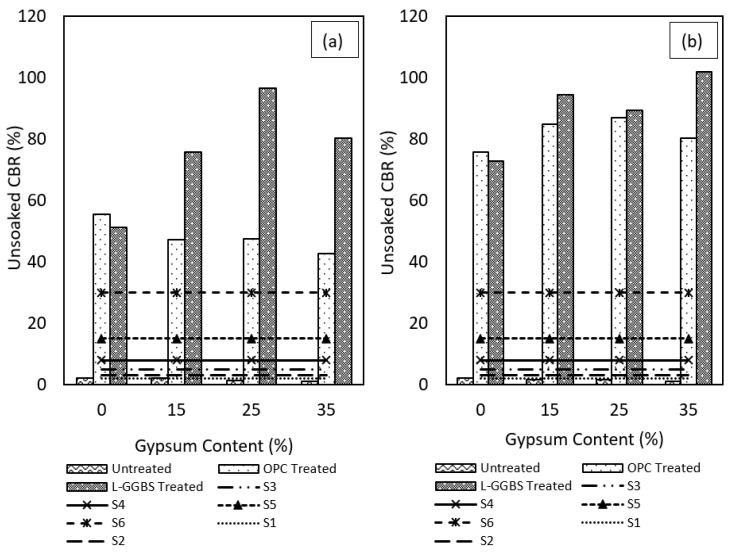
Unsoaked CBR results for OPC and LGGBS treated gypseous soils. (**a**) 7-days cured. (**b**) 28-days cured.

The unsoaked CBR result shows similar behaviour of the treated gypseous soils. Increasing the gypsum content increased the sulphate content and reduced the penetration resistance of the stabilised soils. As with the soaked condition, the 28-days cured samples performed better with higher CBR values as shown in Figure 10a,b.

However, the unsoaked CBR values were lower compared to the soaked values for the treated soils. The results show that soaking the treated samples improved the cementation process in the treated soil since more moisture enhanced the hydration process which increased the strength of the treated soils. On the contrary, the untreated gypseous soil were somewhat better under unsoaked condition than in soaked condition as more water molecules adsorbed within the clay mineral structure caused expansion and reduction in the bond within the kaolin-gypsum matrix which reduced the strength of the untreated soil even further. In terms of the performance of the binders, L-GGBS treatment proved better in both soaked and unsoaked condition compared to OPC treatment.

The performance of the binders has also been compared with standard requirements for subgrade strength prescribed as S1, S2, S3, S4, S5, and S6 as stipulated in [74]. The untreated gypseous soils obviously falls short of the minimum 2.5% CBR for both soaked and unsoaked conditions. Although both OPC and L-GGBS stabilised samples all exceed the subgrade strength requirements, the CBR of the OPC-treated soils might easily depreciate considering the high swell potential of the OPC-treated gypseous soils when exposed to water ingress which can lead to early pavement failures under service conditions. The L-GGBS treated samples meet these requirements without appreciable swelling and is therefore encouraged as a viable alternative to OPC-treated high sulphate-bearing sub-grade.

### 3.6. Microstructural Characteristics

**Figure 11:**
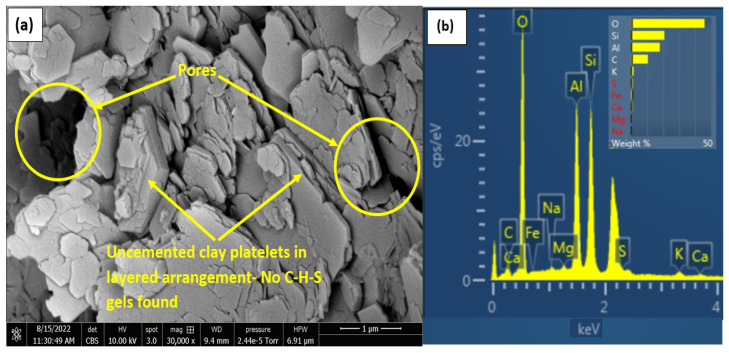
SEM and EDS result of untreated 0%Gypseous soil (Pure Kaolin). (**a**) SEM and (**b**) EDX.

The morphological characteristics and oxide compositions of the treated and untreated gypseous soils have been studied by SEM and EDX analyses. The micrograph of the compacted untreated kaolin (0% gypsum) is presented in Figure 11a. The platelets were found to be uncoated and uncemented as expected, due to non-availability of binding products. The EDS shows spikes in alumina and silica with very low CaO peaks depicting insufficient calcium for development of C-S-H gels. The weak bonding characteristic is mainly from cohesion that exists between the clay platelets in the presence of moisture. An increase in gypsum content from 0% to 35% resulted in slightly increased cementation even for the untreated samples. This is due to a higher calcium content available to react with the Al-Si ions in the clay. The EDX oxide chart also supports this conclusion with a spike in calcium for the untreated non-gypseous soils with increasing gypsum content. Additionally, EDX results shows no spikes in sulphate concentration for the pure kaolin as expected and as shown in Figure 11b. The micrograph of the untreated non-gypseous kaolin was studied to compare changes in the treated soil morphology with that of the untreated non-gypseous soil.

**Figure 12:**
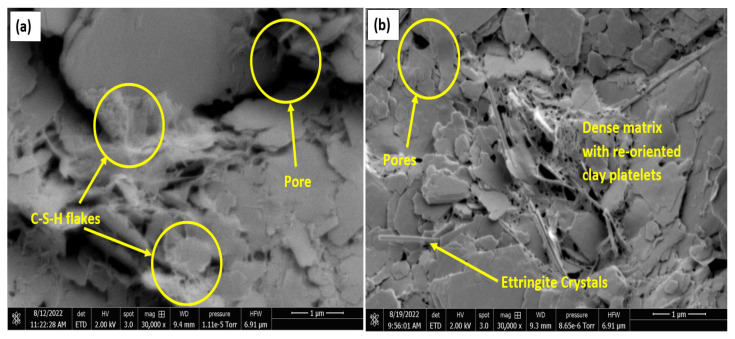

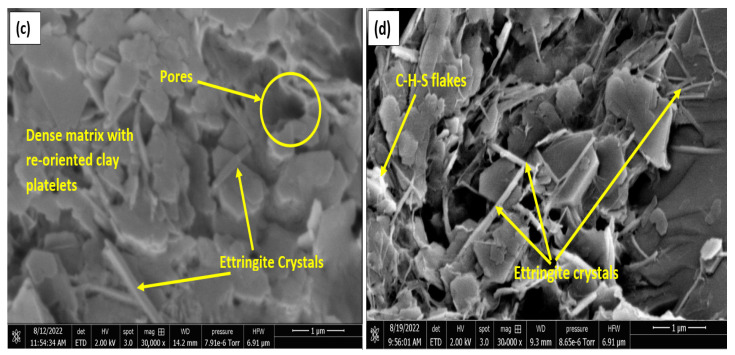
SEM Results of 7-days cured OPC and LGGBS treated gypseous samples. (**a**) OPC-treated 0%Gypsum. (**b**) LGGBS treated 0%Gypsum. (**c**) OPC-treated 35%Gypsum. (**d**) LGGBS-treated 35%Gypsum.

Upon treatment with OPC, an increase in formation of C-S-H compounds is observed in the matrix, as shown in Figure 12a, which results in a better interlock of the particles with reduced pores and increased strength. The LGGBS treated non-gypseous soil showed lower pores as seen in Figure 12b. The non-gypseous soil treated with 12% OPC showed more pores with relatively isolated agglomeration of kaolinite plates with C-S-H gel flakes seen on the plates as shown Figure 12a. When compared to Figure 12b, it is clearly seen that the morphology is characterised by a denser matrix, the kaolinite plates are not seen to be as isolated as in the OPC-treated 0% gypseous soil. This suggests a sharp distinction in both treatments even at the 0%gypsum content. For the treated gypseous soils, the micrographs show that a more sealed soil-binder phase is achieved with the addition of gypsum. While the higher gypseous samples had early ettringite crystal formation, as expected, due to the increased reaction between sulphate ions from gypsum and the tricalcium aluminate (C_3_A) in OPC which seeks to slow down the setting rate [81].

**Figure 13:**
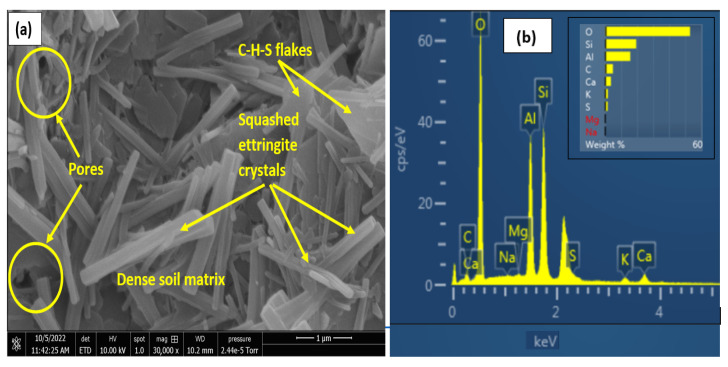

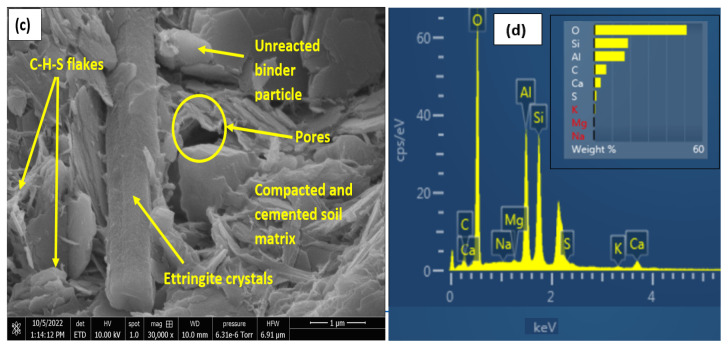
SEM and EDX results of 28-days cured LGGBS-treated gypseous samples. (**a**) SEM of 15%Gypseous soil. (**b**) EDX of 15%Gypseous soil. (**c**) SEM of 35%Gypseous soil. (**d**) EDX of 35%Gypseous soil.

**Figure 14:**
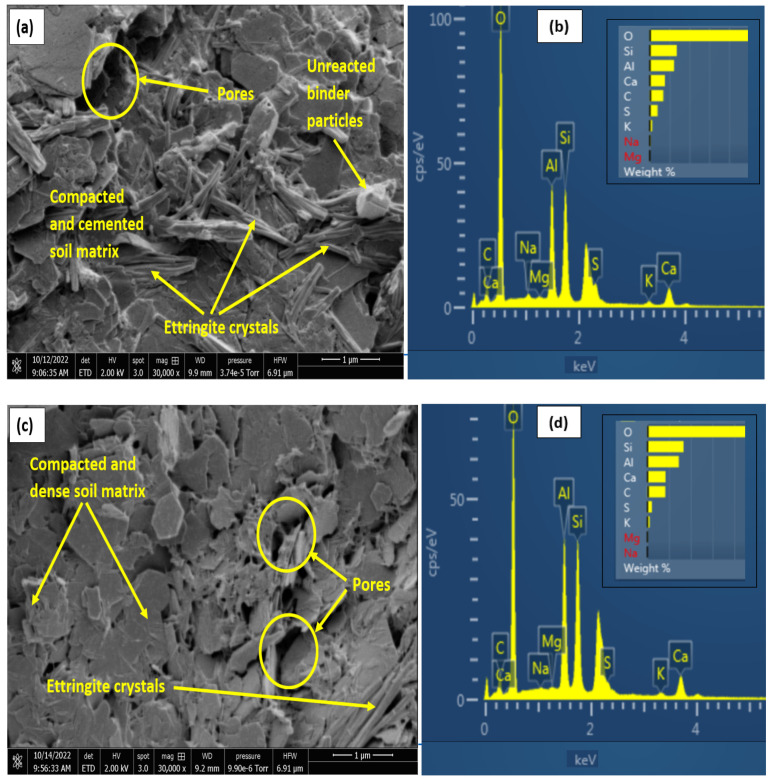
SEM Results of 28-days cured OPC treated Gypseous samples. (**a**) SEM of 15%Gypseous soil. (**b**) EDX of 15%Gypseous soil. (**c**) SEM of 35%Gypseous soil. (**d**) EDX of 35%Gypseous soil.

For the 7-days cured samples, the samples with 35% gypsum content showed a higher compactness, as shown in Figure 12c,d, which would reduce the swell and water absorption. This characteristic aligns well with the swell curves shown earlier. Earlier research work [27,48] have highlighted the fact that more gypsum, above a critical percentage, led to increased cementation and resulted in lower water absorption and expansion. Furthermore, for the treated samples, SEM and EDX results at 28-days curing, as seen in Figure 13 and Figure 14, show more binder flakes and crystals synonymous with long-term curing.

The micrographs also show a simultaneous increase in ettringite crystals with curing duration for both treatments with increasing sulphate content.

However, the 15% gypseous samples were observed to have more ettringite crystals than the 35% for both treatments resulting in the adverse expansive effects as seen in the swell curves. The LGGBS treated samples showed higher percentage of ettringite crystals compared to the OPC samples due to the high alkaline soil-water environment created by the lime. However, the ettringite crystal appeared to be flat and shorter when compared with the more common longer cylindrical prism-shaped crystals in the OPC treated samples. Variations in ettringite crystal shapes have been observed in different sulphate-bearing clays with different additives [82,83]. The higher ratio and random orientation of the ettringite needles seem to act like micro reinforcements which interlocked the clay particles, thereby raising the shear strength of the LGGBS treated samples above the OPC treated samples. The L-GGBS treated soils appeared to be more orientated to accommodate the ensued swell from the ettringite minerals, thereby leading to lower volume change. This mechanism of suppressing volume instability has also been reported in other studies [57].

## 4. Conclusions

The physico-mechanical and microstructural properties of L-GGBS and OPC treated sulphate-bearing soils have been studied with a view to evaluating key parameters of the treated soil relating to pavement design. From the study, the following conclusions have been reached.

1. The use of L-GGBS and OPC treatment are both effective in improving the compressive strength of gypseous soils. However, L-GGBS treated soils showed higher strength than OPC-treated samples for both 7 and 28 days across all dosages of gypsum contents.
2. OPC-treated sulphate-bearing soils are prone to excessive swelling which could pose high risk to light-weight structures such as road pavements, utilities, and some simple shallow foundations due to significant heave upon water ingress. On the other hand, L-GGBS treatment is effective in suppressing sulphate-induced heave for pavement application. The presence of expansive minerals such as ettringite in sulphate-bearing soil poses more concern with OPC than with lime and GGBS. The addition of GGBS appears to modify the cylindrical ettringite needle-like crystals into a squashed cylinder and might have contributed to reducing the expansive tendency of the crystals in the presence of water.
3. The UCS and stiffness modulus of gypseous soils could be improved by treating with L-GGBS combination. An early gain in UCS and stiffness of L-GGBS treated gypseous soils exceeding OPC treated samples suggests that using L-GGBS is a preferable option for rapid strength gain on sites characterised by highly sulphated soils.
4. The CBRs of OPC and L-GGBS-treated soils are enhanced following 96h soaking and could be related to enhanced hydration reaction in the presence of water. Adequate curing is required for stabilised soils to achieve optimal performance as sub-base and sub-grade materials. However, at 7-days curing, the CBR of L-GGBS treated gypseous soil meets the strength requirement for stabilised road-base and sub-base application even at high gypsum content. This indicates that L-GGBS is effective in enhancing the mechanical properties of highly sulphated soils.

## Figures and Tables

**Table 1:** Chemical composition of materials.

Oxide	Kaolin	Gypsum	Lime	GGBS	OPC
	(%)	(%)	(%)	(%)	(%)
SiO_2_	49	10.93	1	34.1	18.84
Al_2_O_3_	36	2.88	0.3	13	4.77
Fe_2_O_3_	0.75	1.16	0.5	0.51	2.87
CaO	0.06	26.32	94	39	61.49
MgO	0.3	-	2	9.5	3.54
K_2_O	1.85	0.83	-	0.5	0.02
TiO_2_	0.02	0.15	-	1.3	0.26
Na_2_O	0.1	0.18	-	0.3	0.57
SO_3_	-	34.70	-	0.3	3.12
Mn_2_O_3_	-	-	1.2	0.7	0.05
LOI	12	19.80	28.79	1.9	4.3

**Table 2:** Gypseous soil classification [50].

Gypsum Content (%)	Classification
0–0.3	Non gypseous
0.3–3	Very lightly gypseous
3–10	Slightly gypseous
10–25	Moderately gypseous
25–50	Highly gypseous

**Table 3:** Atterberg properties of artificially synthesised gypseous soils.

%GYP	PL	LL	PI
0	33	50.5	17.5
15	33	49.6	16.6
25	34	47.6	13.6
35	37	46.2	9.2

**Table 4:** Sample mix compositions.

Grouping	Sample Mix Composition	GYP (wt.%)	OPC (wt.%)	L (wt.%)	GGBS (wt.%)	Moist. Cont. (%OMC)	Curing (Days)
OPC Treated	K + 0%GYP + 12%OPC	0	12	0	0	1.1	7 and 28
	K + 15%GYP + 12%OPC	15	12	0	0	1.1	7 and 28
	K + 25%GYP + 12%OPC	25	12	0	0	1.1	7 and 28
	K + 35%GYP + 12%OPC	35	12	0	0	1.1	7 and 28
L-GGBS Treated	K + 0%GYP + 4%L + 8%GGBS	0	0	4	8	1.1	7 and 28
	K + 15%GYP + 4%L + 8%GGBS	15	0	4	8	1.1	7 and 28
	K + 25%GYP + 4%L + 8%GGBS	25	0	4	8	1.1	7 and 28
	K + 35%GYP + 4%L + 8%GGBS	35	0	4	8	1.1	7 and 28
Untreated	K + 0%GYP	0	0	0	0	1.0	7 and 28
	K + 15%GYP	15	0	0	0	1.0	7 and 28
	K + 25%GYP	25	0	0	0	1.0	7 and 28
	K + 35%GYP	35	0	0	0	1.0	7 and 28

**Table 5:** Swell severity classification.

Swell (%)	Classification
0	No swell
0–0.1	Negligible swell
0.1–0.5	Light swell
0.5–1.0	Medium
1.0–2.0	Strong swell
Above 2.0	Very strong swell

## Data Availability

Data can be obtained from corresponding author upon reasonable request.
